# Yield Performance, Laying Behaviour Traits and Egg Quality of Purebred and Hybrid Hens Reared under Outdoor Conditions

**DOI:** 10.3390/ani10040584

**Published:** 2020-03-31

**Authors:** Chiara Rizzi

**Affiliations:** Department of Agronomy, Food, Natural Resources, Animals and Environment—University of Padova, 35020 Legnaro (PD), Italy; chiara.rizzi@unipd.it

**Keywords:** poultry, local breed, yield performance, laying behaviour, egg quality

## Abstract

**Simple Summary:**

Nowadays, the consumer has a choice of table eggs depending on rearing system, egg size, eggshell and yolk colour, produced by a few worldwide hybrid strains. Many countries have a historic poultry tradition, with breeds used for domestic egg production. In Northern Italy, in the Po river plain, a relevant part of the maize production was used also for poultry feeding and many breeds existed in the last centuries. At present, the Veneto region shows an important poultry biodiversity with many breeds, whose egg production traits are not well known. A comparison between purebred and hybrid hens throughout the laying period was carried out in order to study yield performance, laying behavioural traits and egg quality. The knowledge of the variation of these traits is useful for managing the breeding of these genotypes and the offer to the consumers. The local breeds showed differences from hybrid hens both for yield performance and laying behaviour and for both egg size and external and internal quality.

**Abstract:**

This study compared the yield performance, laying behavioural traits and egg quality of purebred and hybrid hens (from 28 until 44 weeks of age, considering four periods) reared under outdoor conditions. The four genotypes were reared on the same trial station, on four areas (one genotype/area), and under the same environmental conditions from hatching until the end of the trial. Italian dual-purpose purebred (Ermellinata di Rovigo—ER and Robusta maculata—RM) and hybrid (Hy-Line Brown—HB and Hy-Line White 36—HW) hens (flock size: 70 birds/genotype) were allowed outdoors (4 m^2^/bird, good pasture during the growing period and poor pasture throughout the laying period, according to the season) and indoors (0.20 m^2^/bird, five birds/individual nest) and fed commercial feed. Significant (*p* < 0.01) differences among genotypes were found. The hybrids showed a higher laying rate and hen-day edible egg mass, and a lower body weight than the purebreds. Broken and out-of-nest egg% were higher in RM and HW than ER and HB, respectively. Double-yolk egg% was higher in hybrids than in purebreds. The eggshell colour varied among brown eggshell ER, RM, and HB. The ER showed the lowest shape index. With aging, the yolk to albumen ratio linearly increased in all groups, eggshell% changed in ER, HW, RM (cubic) and in HB (linear). The purebreds showed meat spots% higher than blood spots; HW showed the lowest total inclusion%. In conclusion, according to an egg scoring evaluation (egg weight = medium-large size, yolk to albumen ratio = 0.5, total inclusions = none), HW showed a higher quality than HB and RM, and ER was intermediate. The RM hens showed the highest% of defective eggs, especially for overcrowding at nest, HB showed the lowest. Under outdoor conditions the laying behaviour of the purebred hens and the nest management are important factors for the saleable egg rate.

## 1. Introduction

Throughout the last fifty years many traits of poultry eggs have been modified and improved by genetic selection to satisfy the requirements of consumers and a food industry which uses egg albumen and yolk for many purposes [1]. Eggs of hybrid hens differ in eggshell colour, weight and percentages of yolk and albumen according to the strain, but the highest differences exist between hybrid and purebred genotypes [2,3,4,5]. Purebred hens are currently a small part of the worldwide population of reared hens since they are still widely reared in less developed countries [6]. The importance of purebreds is now increasing in industrialized countries as people and rural tourism become more interested in typical food products, with particular characteristics that allow them to distinguish a farm product from an industrial product [4,7]. According to the animals’ welfare goals, outdoor rearing conditions allow the hens to better perform their behavioural traits in comparison to intensive production [8,9] and the farms involved in tourism and animal welfare usually rear chickens and hens under outdoor and pasture conditions.

In outdoor conditions the hen strain and the management of the birds constitute important steps of the productive cycle. In fact the hen genotype mainly affects the oviposition performance and the egg weight and component proportions; behavioural responses of the birds are also important as well as changes in the quality of the eggs throughout the productive cycle. Regarding this last aspect, it is important to stress that the laying period begins at pubertal age that is reached by the animals at different ages and body weights according to the genotype [10,11,12]. Furthermore, the beginning of the productive period and the length of the cycle will depend on the environmental conditions where the breeding activity is performed, on the physiological responses of the birds in terms of body growth and conditions and on the egg production, in terms of the egg mass and quality. In Northern Italy, considering the slow-growing local breeds, the farms usually manage the first period of the laying phase throughout the last six months of the year when the birth and growing period of the chicks occurs at the beginning of the year, or throughout the autumn and winter months, when the birds are hatched in spring. 

In this work a trial was carried out on two Italian breeds and two hybrid genotypes of hens reared under outdoor conditions to monitor changes of yield performance, some laying behavioural and physiological responses of the birds and some egg quality traits throughout the first phase of oviposition. 

## 2. Materials and Methods 

### 2.1. Ethical Statement

The research used eggs produced by hens coming from a rearing farm of the Veneto region, according to the principles stated in EC Directive 86/609/EEC.

### 2.2. Genotypes and Rearing Conditions of the Hens

The eggs used for the trial came from two Italian breeds, Ermellinata di Rovigo (white plumage and black with white edge hackle and saddle; black main tail feathers and white primaries with black extremity,—ER) and Robusta maculata (silver plumage with black tail and white breast feathers, and white primaries with black extremity—RM) and from two commercial hybrid strains, Hy-Line Brown (brown plumage and brown eggshell—HB) and Hy-Line W-36 (white plumage and white eggshell—HW). The Italian breeds are dual-purpose (meat and egg) and slow-growing local genotypes. They were created in Veneto (Italy) during the 1950s: ER (brown eggshell) was created using Sussex and Rhode Island breeds, and RM (brown eggshell) originates from Brown Orpington and White America breeds [13]. 

The hens of each genetic group were reared on the same farm of the Veneto region in Northern Italy, from hatching to pubertal age and throughout the laying period. The newly hatched chicks were kept indoors during the first 4 weeks of life, on litter, under infrared radiation lamps, at an environmental temperature decreasing from 32 to 24 °C. At 2 months of age the birds were given free access to outdoor spaces from spring until autumn. Each genotype (ER, HB, HW: 70 hens and RM: 60 hens) had free access to indoor (0.20 m^2^/bird) space, mainly used for laying eggs, on rainy days and at night, and outdoor (4 m^2^/bird) space for pasturing, where they stayed throughout the day; the area (indoor and outdoor) available to each genotype was divided by netting. The pasture (*Lolium* and *Festuca* prevailing with *Poa*, *Trifolium*, *Taraxacum* and other species) condition was good until the end of spring, throughout the growing period and until the pubertal age, and then it became poor because of the scarcity of rain, eating grass sprouts and walking by the hens, factors that did not allow the grass to regrow. During the laying period the birds showed similar behaviour regarding the use of their outdoor area. In the indoor spaces the floor was covered by a mixture of straw and wood shavings; on the floor, individual nests (30×45 cm, 60 cm height, 1 nest/5 hens) and perches were available to the birds. The animals were given ad libitum a first commercial feed from birth to 16 weeks of age (chemical composition, % as-fed basis: crude protein = 19.4, metabolizable energy = 11.8 MJ/kg) and a second commercial one for laying hens throughout the pubertal age and laying period (17-44 weeks of age) in pelleted form (chemical composition, % as-fed basis: crude protein = 17.0, fats = 5.0, Ca = 3.9 and P = 0.7, lysine = 0.9, methionine = 0.3, metabolizable energy = 11.9 MJ/kg). Feeding, rearing conditions (temperature, photoperiod), and prophylaxis procedures were the same for all groups from the time of hatching until the end of the testing period.

The tested laying period started at 28 weeks of age and lasted until 44 weeks. Throughout the trial the environmental temperature and the relative humidity levels changed according to the season, from summer (28–35 weeks of age: 24 °C, minimum 14 °C, maximum 27 °C) to autumn (36–44 weeks of age: 15 °C, minimum 8 °C, maximum 18 °C). Throughout the laying period the photoperiod was 16L:8D, initially natural according to the seasons and the geographical location of the trial station (Northern Italy) and then it was complimented by artificial light inside the rooms where the birds spent the night. 

### 2.3. Data Collection

Egg production was checked daily during the experimental period. Throughout the trial, on the total daily egg production of each genotype, the number of defective eggs was recorded, considering behavioural defects (dirty eggs, broken eggs, eggs laid outside the nest) and physiological defects (double-yolks, rough eggshell, thin eggshell, bloody eggshell); furthermore, twice/week, the mean daily egg weight (average based on total daily eggs considering samples of 30 eggs as maximum per genotype) was checked for each group. 

Throughout the laying period, from each genetic flock the hen-day egg production (number of eggs/number of live hens × 100) was checked and the hen-day egg mass (yolk and albumen weight) was calculated as hen-day egg production (%) × daily egg yolk and albumen weight (g). The defective egg rate was also calculated as [total hen-day egg production (g) − non defective hen-day egg production (g)]/total hen-day egg production (g) × 100. At 43 weeks of age, the shell colour on 1d-egg was tested by a colorimeter (Chroma meter CR 300 (Minolta Co Ltd., Osaka, Japan), using the CIE [14] scale: the L, a* and b* values reflect lightness (0 = black, 100 = white), redness (-100 = green, 100 = red) and yellowness (−100 = blue, 100 = yellow), respectively. The length (along the longitudinal axis) and width (along the equatorial axis) of the eggs were measured by callipers (0.01 mm) and the shape index as the ratio between them × 100 [15] was also calculated. The egg surface area and volume were calculated following the formula [16]:

SA = (0.9658 × W/L + 2.1378) × L × W and V = 0.525 × L × W^2^

where SA = surface area (cm^2^), V = volume (cm^3^), W = width (cm), L = maximum length (cm).

At regular 4-week intervals (31, 35, 39 and 43 weeks of age) samples of 20-30 eggs (depending on the daily production of each genotype) from a whole day’s production per each genotype were collected, excluding the defective eggs (double-yolk, abnormal shell). The eggshell was broken along the equatorial axis and the yolk and albumen were put on a glass plate making it possible to detect all inclusions, blood and meat spots, respectively, by means of a mirror placed under the glass. Small size inclusion was given 1 point, medium and large size inclusion was given 2 and 3 points, respectively. Yolk was manually separated from the albumen, weighed and the albumen weight was calculated as the difference between the weight of the egg and the sum of the weight of yolk and eggshell (after drying at 50 °C for 12 h).

For an overall classification of the eggs, a total score was obtained by adding together the individual scores based on three main quality characteristics (at 31, 35, 39 and 43 weeks), as follows: egg weight = 1 (≥53 and <73 g), 0.5 (<53 g and ≥73 g); yolk to albumen ratio = 1 (0.5), 0 (<0.5 and >0.5); blood and meat spots = 1 (0 inclusions), 0 (1–3 size inclusions). 

At 44 weeks of age a sample of hens per each group (40 hens per ER, HB and HW and 35 hens per RM) was weighed.

### 2.4. Statistical Analysis

The body weight (at 44 weeks), the hen-day egg production and the defective egg rate (29-44 weeks), the eggshell colour, the egg shape index, the surface area to volume ratio (at 43 weeks), the egg weight and the hen-day yolk and albumen mass (at 31, 35, 39, 43 weeks) were evaluated by ANOVA considering genotype as main effect using the GLM proc of SAS (SAS, Institute, Cary, NC, USA).

The data on yolk, albumen and eggshell percentage (at 31, 35, 39, 43 weeks) for each genotype were evaluated by ANOVA considering age as main effect using the proc GLM of SAS. Significant differences among least squared means were tested using Tukey’s test. For testing linear, quadratic and cubic trends, contrast statements were done using orthogonal polynomial coefficients. For the total score of the eggs the NPAR1WAY proc of SAS, considering genotype as main effect, and the Kruskal-Wallis’s test for detecting significant differences were performed.

For testing significant differences on the percentages of the external (dirty eggs, broken eggs, eggs laid outside the nest, rough eggshell, thin eggshell, bloody eggshell) and internal (double-yolk eggs, blood and meat spots) egg defects Chi squared test was used.

## 3. Results and Discussion

### 3.1. Overall Yield Performance

In Table 1 the effect of genotype on yield performance throughout the laying period is shown. 

The purebreds showed body weights higher (*p* < 0.01) than those of hybrids, and RM and HB were higher (*p* < 0.01) than ER and HW, respectively. The hen-day egg production, indicated as oviposition rate, showed an opposite trend and significant (*p* < 0.01) differences were seen only between the purebreds and the commercial strains. The RM showed the highest (*p* < 0.01) defective egg rate, ER and HW were similar and higher (*p* < 0.01) than HB. The body weight and meat production is not the main trait for commercial high-yielding layer hybrids, but for the native dual-purpose breeds meat production should be considered along with egg production. 

The original idea behind creating these dual-purpose breeds was to create hens with good muscle development. Nowadays, alternative hybrids, such as modern dual-purpose hens, have been created for increasing socio-ethical and legal concerns [17]. In dual-purpose hybrids, the hens should lay a sufficient number of eggs and roosters should show an acceptable fattening performance and thus both sexes gain not only intrinsic, but also economic value [17]. As far as the feed efficiency in egg production is concerned, the comparison between Hy-Line (HLB and HLW) and dual-purpose (ER and RM) hens showed hybrids to be more efficient; for the dual-purpose breeds the feed efficiency concerning body muscle production should also be considered, given that the purebreds showed feed intakes similar to those of hybrids when reared in outdoor conditions similar to those of this trial [18]. This last aspect should be more considered for successfully achieving the aim of a future sustainable egg production and preservation of biodiversity. The result on the defective egg rate, which is comprehensive of the damaged eggs, is important for breeders and for market purposes; in particular the lower saleable RM and ER egg quantity needs to be more and deeply considered as further shown and discussed, given the significant differences resulted between the purebred hens and the hybrids. 

### 3.2. Egg Weight and Daily Edible Egg Mass

**Figure 1:**
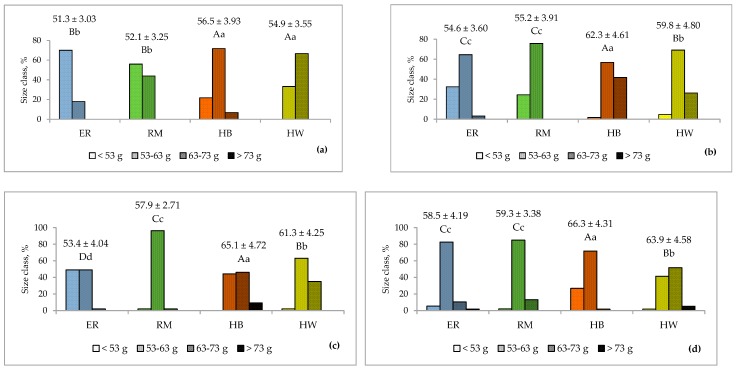
Effect of genotype ^1^ on the egg weight (lsmeans ± SD, g) and size class at 31 (**a**), 35 (**b**), 39 (**c**) and 43 (**d**) weeks of age of the hens. Different letters among genotypes indicate different values for the egg weight (lsmeans). a, b, c, d: *p* < 0.05; A, B, C, D: *p* < 0.01. ^1^ Genotype: ER—Ermellinata di Rovigo; RM—Robusta maculata; HB—Hy Line Brown; HW—Hy Line White 36. Observations (*n*) per age (at 31, 35, 39, 43 weeks): ER (60, 62, 49, 57); RM (50, 37, 52, 53); HB (60, 60, 54, 60); HW (60, 65, 54, 58).

In Figure 1 the comparison of the egg weight among the four groups for each age is shown. Until 31 weeks of age (Figure 1a), the two purebreds showed similar egg weights, significantly lower (*p* < 0.01) than those of the two hybrids. Thereafter (Figure 1b–d), HB showed values higher (*p* < 0.01) than those of HW and the two purebreds showed lower (*p* < 0.01) weights than those of HW; ER was similar to RM, with an exception at 39 weeks (Figure 1c), when it was lower (*p* < 0.01). As far as the egg weight for marketing is concerned, the eggs are classified following the EC Regulation No 589 of 23 June 2008 [19], as small (<53 g), medium (≥53 g and <63 g), large (≥63 g and <73 g) and very-large (≥73 g) size.

**Figure 2:**
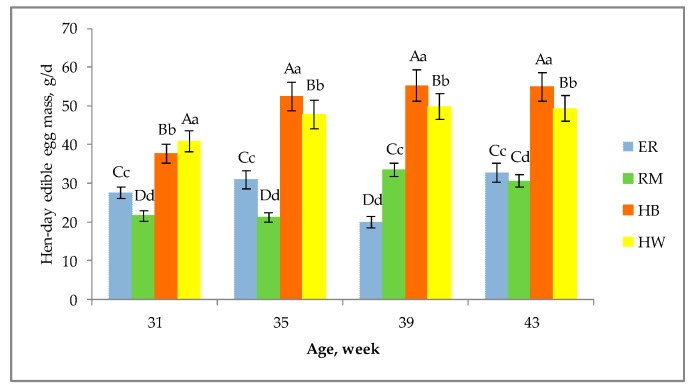
Effect of genotype ^1^ on the hen-day edible egg mass (lsmeans ± SD) according to the age of the hens. Different letters among columns at each age indicate different values. a, b, c, d: *p* < 0.05; A, B, C, D: *p* < 0.01. ^1^ Genotype: ER—Ermellinata di Rovigo; RM—Robusta maculata; HB—Hy Line Brown; HW—Hy Line White 36. Observations (*n*) per age (at 31, 35, 39, 43 weeks): ER (30, 30, 25, 27); RM (25, 22, 30, 29); HB (30, 30, 30, 30); HW (30, 30, 30, 28).

As shown in Figure 1, each genotype showed a certain variability of egg size throughout the laying period considered, as at each age the eggs showed many (from 2 to 4) size classes at different percentages (range from 1.7% in HB and HW at 43 weeks of age to 96% in RM at 39 weeks of age). For ER and RM at the first age (Figure 1a) the egg had a small size (ER = 51.3 g, RM = 52.1 g), and thereafter (Figure 1b–d) a medium size (ER = 55.5 g, RM = 57.5 g); HB eggs until 35 weeks (Figure 1b) showed a medium size (59.4 g) and thereafter (Figure 1c,d) large size (65.7 g) and HW eggs had medium size (58.7 g) followed by large one (63.9 g) at 43 weeks (Figure 1d). Given that the eggs of the purebreds have a lower size than that of hybrids, as stated above, it is also important to evaluate the edible mass produced daily by a hen, with the aim of a useful management of egg production for market purposes. As indicated in Figure 2, the hen-day edible egg mass differed among the groups, according to the ages of the hens. Hybrid hens differed (*p* < 0.01) from the purebreds and between them at all ages: HB edible egg mass was lower (*p* <0.01) than HW at 31 weeks and then higher (*p* < 0.01). The ER production was higher than RM at 31, 35 (*p* < 0.01) and 43 weeks (*p* < 0.05), and lower at 39 weeks (*p* < 0.01). This trend shows that in the purebreds the daily edible egg mass was lower than that of the hybrids as a main consequence of a lower oviposition rate. In fact, from 29 until 44 weeks, the laying rate as shown in Table 1, ranged from 56 to 53% for ER and RM, and from 89 to 87% for HB and HW, respectively.

The two purebreds showed a laying activity lower than the hybrids as at these ages they show a more notable body growth and less nutrient deposition at ovary level and lower protein synthesis in the oviduct with longer periods of not-laying. It is worth remembering that ER and RM, as slow-growing and dual-purpose breeds, showed a lower daily body growth but more prolonged over time than hybrids, which reach a complete muscle growth at about 30 weeks of age [18,20]. The negative correlation existing between reproductive and body growing traits does not allow the dual-purpose hens to achieve the production performance of specialized hybrids. The RM hens showed a delay of onset of laying activity and lower egg mass at 31 and 35 weeks when compared to ER; the opposite trend at 39 weeks indicates that the egg production had fluctuations in comparison to the hybrids, which showed a more constant laying activity at these ages. 

### 3.3. Egg Defects of Behavioural and Physiological Origin

Relevant aspects to investigate on the egg production of purebred and hybrid genotypes under outdoor rearing conditions is the percentage of defective eggs from those suitable for retail, as stated above. For the intensively reared layer hybrid strains, the percentages of defective eggs throughout the laying period and their main causes are known [21,22,23,24]. Little is known about laying behaviour and damaged eggs of dual-purpose hybrids [17,25] and purebred hens reared under outdoor conditions [5]. The number of defective eggs per daily production is very important, given hygiene purposes and the quantity of declassed or lost eggs for market. 

**Figure 3:**
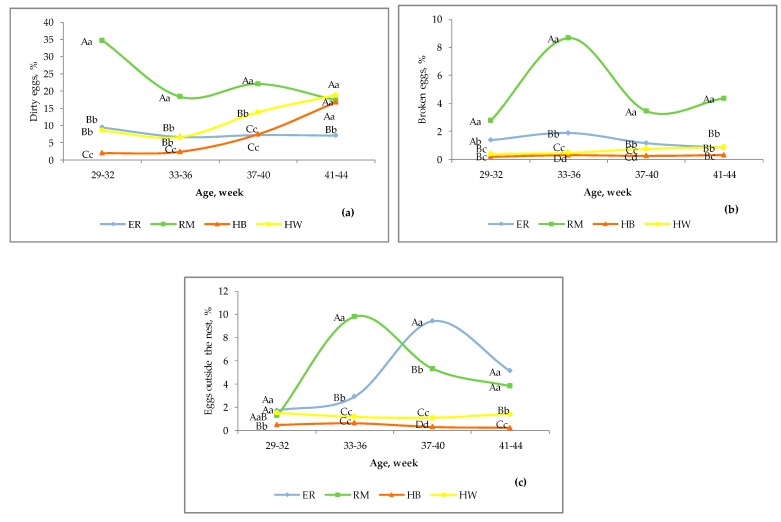
Effect of genotype ^1^ on dirty eggs (**a**), broken eggs (**b**) and eggs outside the nest (**c**) according to the age of the hens. Different letters among lines at each age indicate different values. a, b, c, d: *p* < 0.05; A, B, C, D: *p* < 0.01. ^1^ Genotype: ER—Ermellinata di Rovigo; RM—Robusta maculata; HB—Hy Line Brown; HW—Hy Line White 36. Observations (*n*) per age (29–32 weeks, 33–36 weeks, 37–40 weeks, 41–44 weeks): ER (1450, 1371, 861, 1192); RM (790, 704, 1070, 892); HB (2083, 2271, 2350, 2188); HW (2138, 2565, 2004, 1833). χ^2^ *p*-value per age: dirty eggs (<0.0001 for all the ages); broken eggs (<0.0001 for all the ages); eggs outside the nest (at 29–32 weeks = 0.003, and then <0.0001).

In Figure 3, three laying behavioural causes for the defective egg percentages are summarized. 

In Figure 3a, the dirty egg percentage is shown: a notable differentiation (*p* < 0.01) among the groups was observed until 37–40 weeks, with the highest values for RM, followed by ER, HW and HB. At 41–44 weeks of age the values were similar for the groups, with the exception that ER showed lower (*p* < 0.01) percentages. The RM eggs constantly showed high values, 35% at 29–32 weeks, and thereafter about 20%, ER values were quite stable between 7 and 10%. HW was similar to ER until the II age and then increased to 18%, and HB was stable and lower than 5% until the II age and then gradually increased under 10 and 20%, respectively, at 37–40 and 41–44 weeks. There are many factors responsible for a dirty eggshell, both direct and indirect, induced by behavioural and physiological effects. For RM the highest dirty values checked at 29–32 weeks were due mainly to the breaking of some eggs thereby dirtying the eggshell of the other eggs with the internal constituents: this breed started the laying activity later than the other groups, and they showed gregarious nesting and crowding, which contributed to some eggs breaking. Possible effects of different nest occupation and egg laying circadian rhythms during the day may explain the different response of the genotypes [26]. The percentages observed from 37 until 44 weeks were induced by environmental and weather factors, such as rain and fog, and by the behaviour of the hens before laying. When the level of humidity of the outdoor surface was high, as occurs in Northern Italy during the autumn, the birds that went to the nest after using the outdoor space soiled the eggshell (HB and HW), whereas the hens that stayed in the indoor space before laying had cleaner eggs (ER) and this situation occurred in particular when the oviposition occurred after night. 

In Figure 3b, the percentages of broken eggs are shown. The RM genotype was constantly higher (*p* < 0.01) than the other groups; ER differed (*p* < 0.01) from hybrids, with exception at IV age, and HW was significantly higher than HB after 32 weeks. The RM trend mainly reflects the laying behaviour of the birds and an overcrowding inside the nests, but also other stressing environmental factors may have damaged the eggs [26]. In hens, egg laying in a common nest may be an anti-predator behaviour [27]: the ER and RM hens came from a population reared under outdoor conditions for generations in a regional poultry breeding conservation centre, and thus they may have developed an anti-predator behaviour due to the presence of avian (especially *Corvidae*) and mammalian predators more than the hybrids. Predation in organic and free-range egg production has been recently documented [28] as it may cause yield losses. Other authors reported different patterns of nest use and nest behaviour between conventional layers and dual-purpose hens that seemed to be more affected by nest location than conventional layers [25]. More research is needed for these breeds on the pre-laying and nesting behaviour and hormonal status, factors which may affect the declassed egg rate for a genotype. 

In Figure 3c, the percentages of eggs laid outside the nest are shown. The comparison among genotypes showed different percentages at almost all the ages considered. The RM hens had more eggs laid outside the nest (*p* < 0.01) than ER from 33 until 40 weeks of age; the purebreds were higher (*p* <0.01) than hybrids at almost all the ages, with an exception at I age, when a difference (*p* < 0.05) was detected only in comparison to HB. The HW hens showed a laying behaviour significantly different from that of HB, as shown by higher percentages at almost all the ages (*p* < 0.01). Causes affecting this behaviour during the first weeks of activity may be inexperience, while for the later periods this may be due to possible conditions of stress or disturbance among the birds [8,28]. The group size could be a factor affecting the rate of mislaid eggs by hens, as in presence of the same nesting area per hen a less competition for the nest in larger groups should occur, but the results are not conclusive as observed in hens reared in enriched cages and aviaries [24]. It is unknown whether the different genotypes had a different laying behaviour in relation to the artificial light schedule. 

The data concerning the quality and integrity of the eggshell are important when table eggs are considered: Messens et al. [29] noted that the factors that contribute to the penetration of microorganisms within the egg contents include contamination of the shell surface and cracks in the cuticle and shell. Furthermore, the quality of the cuticle [30] deposited on the shell is also an important attribute that influences bacterial contamination [31]. Abnormal calcification is another attribute that could increase the permeability of the eggshell to microorganisms. Misplaced eggs is an important factor that impairs profitability of an egg laying farm because it is associated with a higher rate of declassed eggs, and work overload due to manual egg collection. 

**Figure 4:**
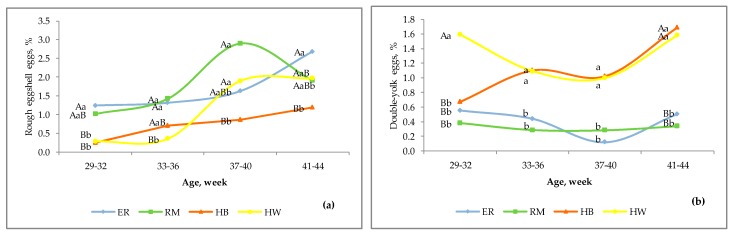
Effect of genotype ^1^ on rough eggshell eggs (**a**) and double-yolk eggs (**b**) according to the age of the hens. Different letters among lines at each age indicate different values. a, b, c, d: *p* < 0.05; A, B, C, D: *p* < 0.01. ^1^ Genotype: ER—Ermellinata di Rovigo; RM—Robusta maculata; HB—Hy Line Brown; HW—Hy Line White 36. Observations (*n*) per age (29–32 weeks, 33–36 weeks, 37–40 weeks, 41–44 weeks): ER (1450, 1371, 861, 1192); RM (790, 704, 1070, 892); HB (2083, 2271, 2350, 2188); HW (2138, 2565, 2004, 1833). χ^2^ *p*-value per age: rough eggshell eggs (<0.0001, 0.002, 0.0001, 0.22); double-yolk eggs (0.0008, 0.04, 0.009, 0.0008).

Figure 4 shows the defective eggs for two physiological causes. The defective eggs with rough shell (Figure 4a) showed an increasing trend according to the age for the four groups, with the most significant (*p* < 0.01) differences between purebreds and hybrids. Until 33–36 weeks, the percentages were under 1.5 for all the groups, and thereafter the purebreds exceeded 2.5% and HW was under 2%. The HB hens showed a more limited increase, reaching 1% on average. 

The eggshell formation and the mineral deposition may be impaired by many factors, and the most relevant factor is the phase of oviposition and the weeks of activity of the oviduct for the synthesis of eggshell membranes and a good deposition of calcium carbonate. These phases are regulated by a physiological status but stress factors such as some environmental conditions and crowding in nests which can cause egg retention within the shell gland, could affect the uniformity of the mineral layer [32].

The double-yolk egg percentages are in Figure 4b. The HW hens showed higher percentages (*p* < 0.01) than those of purebreds throughout the overall production period and they showed more double-yolk eggs (*p* < 0.01) than HB only at I age. This defect is of physiological origin and the hormonal status can affect the ovulation rate in particular at the beginning or at the end of the laying activity according to the genotype and the interaction with lighting [21,33]. The hybrids, selected for an intense oviposition rate, showed similar values and trends from 33–36 weeks, and exhibited an increasing differentiation from the purebreds, with percentages 4–5 times higher. 

**Figure 5:**
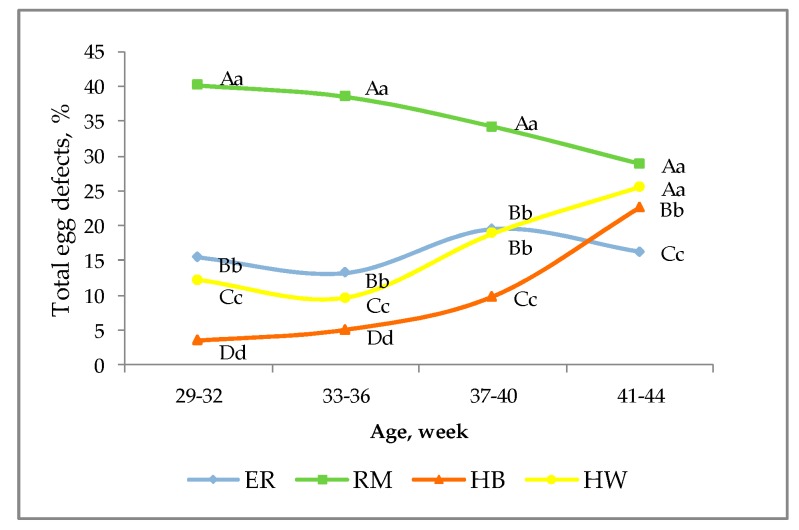
Effect of genotype ^1^ on total main behavioural and physiological egg defects according to the age of the hens. Different letters among lines at each age indicate different values. a, b, c, d: *p* < 0.05; A, B, C, D: *p* < 0.01. ^1^ Genotype: ER—Ermellinata di Rovigo; RM—Robusta maculata; HB—Hy Line Brown; HW—Hy Line White 36. Observations (*n*) per age (29–32 weeks, 33–36 weeks, 37–40 weeks, 41–44 weeks): ER (1450, 1371, 861, 1192); RM (790, 704, 1070, 892); HB (2083, 2271, 2350, 2188); HW (2138, 2565, 2004, 1833). χ^2^ *p*-value: <0.0001 for all the ages.

Figure 5 resumes the percentages of total defective eggs for the behavioural and physiological causes considered in this trial throughout the productive period. 

The four groups were significantly differentiated at all the ages. The RM hens showed more total defective eggs (*p* < 0.01) than the other three genotypes, and decreasing percentages throughout the period ranging from 40 to 29%. At all the ages HW showed higher (*p* < 0.01) values than HB and lower (*p* < 0.01) than ER and an increasing trend from 33 until 44 weeks, when the hybrids were higher (*p* < 0.01) than ER. The HB group showed the lowest percentages, less than 5%, until 36 weeks and then increased reaching more than 15% at the IV age. The results of hybrids are opposite to those checked on the same hen strains reared in an aviary system [26].

The results on the damaged and defective eggs indicate that the highest values are mainly due to nest overcrowding and eggs out of the nest; the effect of bodies and legs on the eggs for overcrowding is more negative regarding heavy hens and individual nests. These results indicate the need of further studies on pre-laying and nesting behaviour to elucidate whether nests of bigger size or collective nests could lower the damaged egg rate, but other factors should also be considered. In fact, as stated by other authors [25] the nest location preferences or the perceptions of nest attractiveness [26,34] as well as the different sensitivity for any environmental stressing factors showed by the hen genotypes should be taken into account.

### 3.4. External Traits of the Eggs

Some external traits of the eggs of the four genotypes are shown in Table 2. The eggshell showed white colour and different shades of brown according to the genotype: the lightness (L) significantly differed among the genotypes with the highest (*p* < 0.01) values for HW with white eggshell, in comparison to the other groups with brown eggshells. The lightness differed among purebreds and HB: ER was higher (*p* < 0.01) than RM and RM was higher (*p* < 0.01) than HB. The redness index (a*) followed the opposite trend, as well as the values of the yellowness index (b*): HW showed the lowest (*p* < 0.01) values, ER was lower (*p* < 0.01) than RM and RM was lower (*p* < 0.01) than HB. The shape index was lower (*p* < 0.01) in ER in comparison to the other three groups. The surface area to volume ratio was higher in ER than in RM (*p* < 0.05), and in HW than in HB (*p* < 0.01); ER had the highest and HB the lowest values, respectively. 

All of these parameters are very important for the processes occurring inside the eggs during the brooding period in fertilized eggs but also for table eggs during storage and for being chosen by the consumer. In fact, although the colour and the shape index as well the texture of the shell do not influence the nutritional value of eggs, these external characteristics are important from a sales and marketing perspective [1,35]. The preference for table eggs with white or brown shells generally has a historical tradition, as in some countries white-shelled eggs and in others brown-shelled eggs are usually commercialized at market [1]. For evaluating the quality of an egg, the consumer firstly considers the external aspect and for brown eggs it is represented by the colour intensity of the shell. Nowadays, the consumer is only familiar with brown or white eggshells, produced by hybrid hens. Even if the consumer does not know the physiological mechanisms for eggshell formation inside the body of the hen, he evaluates the colour uniformity and intensity. Consumers are increasingly concerned with the health implications of food items; hence, egg customers are interested in both the external and internal quality of eggs. Most often, the internal quality of an egg is considered to have a direct correlation with its external features, and the consumer may evaluate an egg and its shell colour from this perspective. The shell colour of commercial table eggs is brown, white or tinted according to the presence of pigments deposited on the cuticle, the outer surface of the shell. On an eggshell from a healthy hen the deposition of pigments, uniformly or spotted, and the colour, such as tint and intensity, depends on genotype and age of the hens [36]. 

The ER and RM eggshells are differently coloured and have a different pattern of deposition of pigments in comparison to HB. A differently coloured eggshell, as well as the egg size, could be an identification element for eggs of different genotypes. 

As indicated by the shape index, the ER eggs are less spherical in comparison to the eggs of the other groups and have a higher surface area to volume ratio. The shape index of eggs of *Gallus gallus domesticus* hens is in the middle of the range of avian eggs which is usually between 65 to 85% [37]. The normal or characteristic shape of the egg is determined in the magnum, but the specific shape of the shell membranes, as determined in the isthmus, has a direct influence on the shell shape [32].

The egg shape may vary but it should limit water loss and also permit gaseous exchange according to a good balance. The shape of an egg should be optimized for the environmental conditions where the breeding activity of the birds occur: in fact the eggs of birds living in deserts, at high altitudes, or at high temperatures have a relatively low shell conductance [38,39]. There is a lower limit on eggshell conductance and permeability, given the need for effective exchange of oxygen and carbon dioxide between the embryo and the atmosphere. For any given volume, a spherical egg has a lower surface area to volume ratio than does an ovoid egg. Therefore, all else being equal, a spherical egg will gain and lose heat more slowly, lose less water, and have lower exposure to solar radiation than will a more elongated egg with the same volume [40]. 

In this trial the ER eggs showed a more ovoid shape than RM as well as HW in comparison to HB, indicating a possible higher water loss during storage, due to a lower eggshell thickness [41]. In domestic chickens, *Gallus gallus*, the relationships between egg shape and shell conductance has not been well explored [39]. Furthermore, it is worth remembering that globular eggs, such as RM and hybrid eggs, were found to be more resistant to breakage [41].

### 3.5. Internal Traits of the Eggs

Table 3 shows the components of an egg throughout the first phase of the productive cycle. The yolk incidence on the egg weight significantly and linearly (*p* < 0.01) increased with age in all the groups, but to a different extent. ER and RM reached the highest values only at 43 weeks of age, whereas HB and HW did it before this age. The albumen incidence linearly (*p* < 0.01) decreased in all the groups, showing significant differences only in ER and HW between 31 and 35 weeks of age. The eggshell incidence showed a cubic (*p* < 0.01) trend in ER, RM and HW, with the lowest values at 35 and the highest at 39 weeks, and then a decrease. Only HB showed a linear trend, with a constant decrease after 31 weeks of age. As a consequence of these changes in the deposition of yolk and albumen, the ratio between them increased linearly (*p* < 0.01) in all the groups, more gradually in ER, RM and HB and more markedly in HW. A yolk to albumen ratio of 0.50 was reached only by purebreds, by ER from 39 weeks and by RM from 43 weeks, whereas hybrids always showed values less than 0.50, HB gave eggs with a ratio < 0.40 and HW > 0.40 only from 35 weeks. 

As known, in commercial strains the yolk weight increases with age [24,42] according to a higher food ingestion and lower request for body growth, but in our conditions in ER it did not change at 39 and 35 weeks of age, possibly due to an interaction between environmental conditions (high temperature), body growth, the laying activity and the hormonal status. Concerning the changes of eggshell incidence throughout the laying period according to the genotype, these may be due to interaction between environmental conditions and the physiological status of the birds: the hens laid eggs with different calcium depositions during each shell formation cycle or diluted the same calcium quantity over a larger surface [24,32] of the eggshell. 

**Figure 6:**
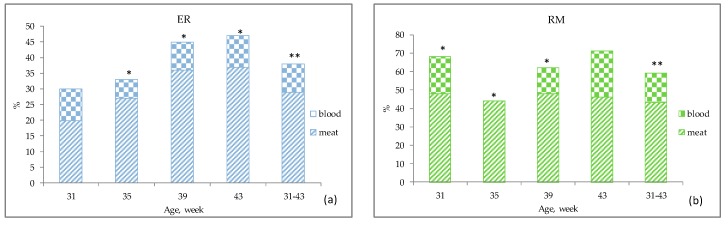

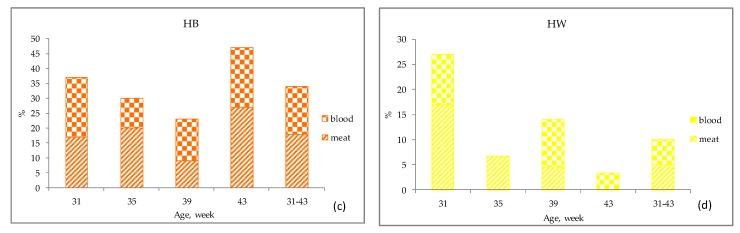
Yolk (blood spots) and albumen (meat spots) quality of each genotype^1^ according to the age of the hens. Different symbols on the columns at each age indicate different values. *: *p* < 0.05; **: *p* < 0.01. ^1^ Genotype: ER—Ermellinata di Rovigo; RM—Robusta maculata; HB—Hy Line Brown; HW—Hy Line White 36. Observations (*n*) per age (at 31, 35, 39, 43 weeks): ER (30, 30, 22, 30); RM (25, 16, 21, 24); HB (30, 30, 22, 30); HW (30, 30, 22, 30). χ^2^ *p*-value per age (at 31, 35, 39, 43 weeks): ER (0.28, 0.04, 0.03, 0.01, total period 0.002); RM (0.03, 0.03, 0.02, 0.13, total period 0.004); HB (0.74, 0.45, 0.64, 0.54, total period 0.99); HW (0.45, not determined, 0.55, 0.32, total period 0.99).

**Figure 7:**
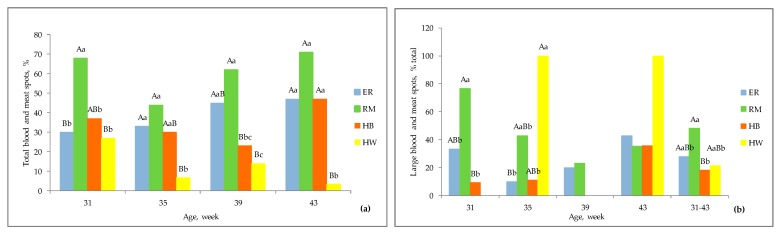
Effect of genotype ^1^ on total blood and meat spots (**a**) and on large (size 2–3) blood and meat spots (**b**) of the eggs according to the age of the hens. Different letters among columns at each age indicate different values. a, b, c: *p* < 0.05; A, B, C: *p* < 0.01. ^1^ Genotype: ER—Ermellinata di Rovigo; RM—Robusta maculata; HB—Hy Line Brown; HW—Hy Line White 36. Observations (*n*) per age (at 31, 35, 39, 43 weeks): ER (30, 30, 22, 30); RM (25, 16, 21, 24); HB (30, 30, 22, 30); HW (30, 30, 22, 30). χ^2^ *p*-value per age (at 31, 35, 39, 43 weeks): total blood and meat spots (0.008, 0.02, 0.004, <0.0001); meat and blood spots (size 2–3) (0.0002, 0.03, 0.55, 0.08, total period 0.01).

The yolk and albumen quality in table eggs may also be evaluated by blood and meat spots (Figure 6 and Figure 7). The purebreds (Figure 6a,b) showed a higher (*p* < 0.01) incidence of meat spots than blood spots throughout almost the entire laying period considered, whereas HB showed a balanced presence of meat and blood spots as well as HW (Figure 6c,d).

Therefore, as indicated in Figure 7a, RM gave eggs with the highest percentage of meat and blood spots, with values ranging from 42 to 70%, similar to those of the other groups with brown eggshell at almost all the ages. The two hybrids were similar at I and III age, otherwise HW showed lower values. In Figure 7b the incidence of meat and blood spots of large size on the total inclusions are represented. RM showed significantly higher percentages, more than 50%, at 31 weeks of age, than ER (*p* < 0.01) and HB (*p* < 0.05) and then the incidence was similar among the groups. The HW hens showed variable percentages throughout the period studied, indicating that the inclusions, when present, were relevant. These results are mainly due to the physiology of reproduction of the hens: at ovulation the rupture of the ovarian follicle at an area different from stigma and residues of the activity of the oviduct for the albumen synthesis can generate blood and meat spots in particular in genotypes with a genetic asset originally not addressed to a high laying activity [21,32].

This is evident in ER and RM genotypes, defined as dual-purpose breeds, and in HB. For this late genotype, the effect of selection on improving these characters was evident especially at IV age. The effect of the production system, such as conventional cages, enriched cages and aviaries did not cause differences for blood and meat spots [24]. 

**Figure 8:**
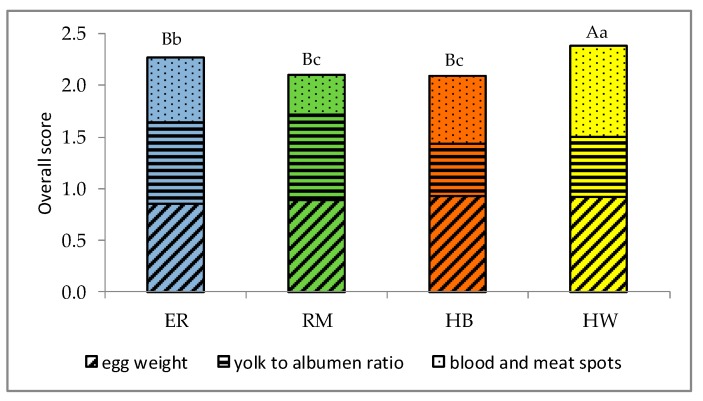
Effect of genotype ^1^ on the overall score of the eggs based on egg weight, yolk to albumen ratio and blood and meat spots. Different letters among columns indicate different values. a, b, c: *p* < 0.05; A, B, C: *p* < 0.01. ^1^ Genotype: ER—Ermellinata di Rovigo; RM—Robusta maculata; HB —Hy Line Brown; HW—Hy Line White 36. Observations (*n*) per each genotype: 112. χ^2^ *p*-value: 0.0002.

An overall evaluation of egg quality is shown in Figure 8. 

For an evaluation on scoring, three main variables of the egg have been included: the weight, the yolk to albumen ratio and the blood and meat spots. Given that some commercial hybrid strains have been selected to have greater egg size and a yolk to albumen ratio < 0.5, as HB and HW showed in Figure 1 and in Table 3, the criterion for the scoring was addressed to a medium-large sized egg, with a yolk to albumen ratio of 0.5, and with no blood and meat inclusions. The HW score was higher (*p* < 0.01) than those of HB and purebreds, ER was higher (*p* < 0.05) than RM.

In addition to this evaluation, the eggshell colour and the shape index characterize the eggs of these genotypes and may be useful for addressing the choice of the consumer.

## 4. Conclusions

The knowledge of the variation of the yield performance and egg quality according to the hen genotype is useful for managing the offer for consumers. Under outdoor rearing conditions, egg production changed according to the physiological and behavioural responses of the hen genotype and to the season. The dual-purpose local breeds showed lower daily egg mass than hybrids, but a higher meat production; the purebred eggs differed from the hybrid eggs also in external and internal quality. According to a score evaluation (egg weight = medium-large size, yolk to albumen ratio = 0.5, total inclusions = none), HW quality was higher than those of HB and RM, and ER was intermediate. The RM hens showed the highest% of defective eggs, especially due to overcrowding; HB showed the lowest one. The laying behaviour of the purebred hens under outdoor conditions and nest management are important factors for determining the saleable daily egg rate.

## Figures and Tables

**Table 1:** Effect of genotype on yield performance of the hens from 29 until 44 weeks of age.

	Genotypes ^1^					
	**ER**	**RM**	**HB**	**HW**	RMSE	*p*-Value
Body weight at 44 weeks, kg	2.6 ^Bb^	3.0 ^Aa^	2.0 ^Cc^	1.7 ^Dd^	0.20	<0.0001
Oviposition, %	56 ^Bb^	53 ^Bb^	89 ^Aa^	87 ^Aa^	13	<0.0001
Defective egg rate, %	16.3 ^Bb^	36.0 ^Aa^	9.9 ^Cc^	17.4 ^Bb^	13.73	<0.0001

Different letters among columns indicate different values. a, b, c, d: *p* < 0.05; A, B, C, D: *p* < 0.01. RMSE = Root Mean Squared Error. ^1^ Genotypes: ER—Ermellinata di Rovigo; RM—Robusta maculata; HB—Hy Line Brown; HW—Hy Line White 36. Observations (*n*) per genotype: oviposition and defective egg rate (111).

**Table 2:** Effect of genotype on the eggshell colour and egg dimensions at 43 weeks of age of the hens.

	Genotypes ^1^					
	**ER**	**RM**	**HB**	**HW**	RMSE	*p*-Value
Eggshell colour						
L	76.9 ^Bb^	67.1^Cc^	60.2 ^Dd^	80.3 ^Aa^	3.51	<0.0001
a*	8.6 ^Cc^	12.3 ^Bb^	17.1 ^Aa^	0.3 ^Dd^	1.92	<0.0001
b*	22.2 ^Cc^	24.7 ^Bb^	29.0 ^Aa^	0.3 ^Dd^	1.92	<0.0001
Shape index, %	72.6 ^Bb^	76.5 ^Aa^	76.1 ^Aa^	77.0 ^Aa^	2.87	<0.0001
Surface area to volume ratio	1.28 ^Aa^	1.26 ^ABb^	1.23 ^Cc^	1.25 ^Bb^	0.0261	<0.0001

Different letters in a row indicate different values. a, b, c, d: *p* < 0.05; A, B, C, D: *p* < 0.01. RMSE = Root Mean Squared Error. ^1^ Genotypes: ER—Ermellinata di Rovigo; RM—Robusta maculata; HB—Hy Line Brown; HW—Hy Line White 36. Observations (*n*): ER (31); RM (31); HB (30); HW (30).

**Table 3:** Effect of age on yolk, albumen, shell percentage and yolk to albumen ratio according to the genotype of the hens.

	Genotypes ^1^			
	ER	RM	HB	HW
Yolk, %				
31 weeks	27.9 ^Cc^	26.6 ^Cc^	23.4 ^Bb^	24.5 ^Bb^
35 weeks	29.8 ^Bb^	28.6 ^AaBb^	24.4 ^AaB^	26.6 ^Aa^
39 weeks	30.1 ^Bb^	28.5 ^Bb^	25.3 ^Aa^	26.6 ^Aa^
43 weeks	31.6 ^Aa^	29.7 ^Aa^	25.2 ^Aa^	27.1 ^Aa^
RMSE	1.76	1.47	1.50	1.46
Component *p*-value	linear <0.0001	linear <0.0001	linear <0.0001	linear <0.0001
Albumen, %				
31 weeks	62.1 ^Aa^	62.4	65.2	65.2 ^Aa^
35 weeks	60.4 ^Bb^	60.7	64.9	63.4 ^Bb^
39 weeks	59.4 ^Bbc^	59.9	64.2	62.6 ^Bb^
43 weeks	58.8 ^Bc^	58.9	64.3	62.6 ^Bb^
RMSE	2.07	1.61	1.66	1.61
Component *p*-value	linear <0.0001	linear <0.0001	linear 0.0118	linear <0.0001
Eggshell, %				
31 weeks	10.0 ^Aa^	11.0 ^ab^	11.4 ^Aa^	10.3 ^ABb^
35 weeks	9.8 ^Bb^	10.7 ^b^	10.7 ^Bb^	10.0 ^Bb^
39 weeks	10.5 ^Aa^	11.6 ^a^	10.5 ^Bb^	10.8 ^Aa^
43 weeks	9.5 ^Bb^	11.3 ^ab^	10.5 ^Bb^	10.3 ^AaBb^
RMSE	0.86	0.91	0.90	0.75
Component *p*-value	cubic 0.0004	cubic 0.0105	linear <0.0001	cubic <0.0001
Yolk to albumen ratio				
31 weeks	0.452 ^Cc^	0.430 ^Cc^	0.361 ^Bb^	0.377 ^Bb^
35 weeks	0.497 ^Bb^	0.476 ^Bb^	0.378 ^AaBb^	0.422 ^Aa^
39 weeks	0.512 ^AaBb^	0.482 ^Bb^	0.397 ^Aa^	0.427 ^Aa^
43 weeks	0.541 ^Aa^	0.512 ^Aa^	0.394 ^Aa^	0.436 ^Aa^
RMSE	0.0476	0.0368	0.0326	0.0339
Component *p*-value	linear <0.0001	linear <0.0001	linear <0.0001	linear <0.0001

Different letters among rows of each genotype indicate different values. a, b, c: *p* < 0.05; A, B, C: *p* < 0.01. RMSE = Root Mean Squared Error. Only the component with the highest and significant *p*-value is shown. ^1^ Genotypes: ER—Ermellinata di Rovigo; RM—Robusta maculata; HB—Hy Line Brown; HW—Hy Line White 36. Observations (*n*) per age (at 31, 35, 39, 43 weeks): ER (30, 32, 25, 27); RM (25, 15, 30, 29); HB (30, 30, 30, 30); HW (30, 35, 30, 28).
